# Supplementary material for: Human arm endpoint-impedance in rhythmic human-robot interaction exhibits cyclic variations
Source: PLoS One. 2023 Dec 14;18(12):e0295640. doi: 10.1371/journal.pone.0295640 (PMC10721195; doi:10.1371/journal.pone.0295640)
Supplement: S1 Data — (PDF) [file pone.0295640.s001.pdf]

**K** (N/m)						
	Experiment P			Experiment H		
User ref	φ_1	φ_2	φ_3	$h_1^*(\varphi_1)$	$h_3^*(\varphi_1)$	Performance group
000	151.01	194.28	163.60	139.05	159.31	e_3
008	135.54	191.70	196.69	115.68	265.03	e_2
029	95.06	77.88	294.70	52.41	110.39	e_1
041	180.04	138.76	218.04	151.49	293.19	e_3
050	106.73	147.98	327.81	72.26	88.47	e_1
113	77.49	106.49	122.68	88.54	86.11	e_2
160	240.15	343.64	266.96	85.04	134.10	e_1
194	95.81	116.49	165.49	75.91	76.61	e_2
208	107.34	122.58	119.41	93.67	82.80	e_1
211	445.13	106.65	140.24	117.58	163.57	e_2
278	82.55	95.86	145.14	57.51	159.55	e_1
311	96.44	174.33	163.08	56.62	82.44	e_2
353	53.44	249.60	131.58	113.80	84.52	e_1
364	65.91	211.43	219.79	61.38	62.61	e_2
378	67.05	267.46	200.63	71.73	69.60	e_3
388	127.66	131.71	147.54	113.12	99.65	e_2
418	57.17	118.11	168.03	76.47	44.89	e_2
456	179.05	112.79	113.69	88.32	191.93	e_1
495	88.43	94.38	135.38	75.98	110.25	e_2
546	109.86	138.34	178.74	103.44	109.99	e_1
548	95.46	61.91	249.80	101.38	73.26	e_3
555	188.24	191.52	139.13	131.83	150.05	e_2
573	71.06	256.51	220.83	145.24	98.14	e_3
640	73.26	105.75	219.50	134.29	99.28	e_1
661	70.78	75.90	177.00	127.53	97.58	e_2
666	81.29	163.73	216.48	101.68	90.12	e_2
710	222.84	289.48	276.01	400.60	220.25	e_2
740	72.38	162.80	135.20	156.69	58.54	e_1
841	136.12	131.96	190.60	94.39	93.57	e_1
851	199.08	224.35	362.73	148.43	120.82	e_1
922	91.30	147.44	128.28	104.94	154.05	e_1

Table 1: Estimated stiffness K for each participants, according to the experimental conditions

**B** (N.s/m)						
	Experiment P			Experiment H		
User ref	φ_1	φ_2	φ_3	h_1^* (φ_1)	h_3^* (φ_1)	Performance group
000	15.00	12.86	10.15	16.39	16.75	e_3
008	9.22	12.24	10.34	10.49	13.20	e_2
029	9.35	11.04	9.10	10.02	12.08	e_1
041	10.20	14.44	10.83	12.21	12.08	e_3
050	12.24	13.78	11.08	10.60	10.02	e_1
113	8.30	12.09	7.90	5.70	10.39	e_2
160	11.84	14.76	12.31	12.95	9.78	e_1
194	8.32	8.76	8.41	9.77	5.33	e_2
208	9.17	11.07	8.86	8.88	11.87	e_1
211	10.20	9.41	7.99	11.52	16.48	e_2
278	12.40	10.86	6.69	9.52	10.21	e_1
311	8.81	10.61	8.66	9.18	9.26	e_2
353	9.60	15.34	11.88	10.43	9.79	e_1
364	7.03	14.33	17.07	8.69	7.07	e_2
378	11.21	17.51	8.85	11.52	12.53	e_3
388	12.94	10.61	8.63	13.20	11.59	e_2
418	10.70	11.90	10.05	9.85	3.54	e_2
456	14.90	8.47	8.94	14.63	14.96	e_1
495	8.74	7.92	8.77	8.33	11.34	e_2
546	10.41	11.33	10.51	3.87	8.69	e_1
548	8.82	8.46	8.85	7.50	4.77	e_3
555	8.68	9.92	6.85	5.35	9.60	e_2
573	9.31	15.27	13.50	5.04	13.13	e_3
640	8.91	10.16	11.79	9.66	9.05	e_1
661	8.51	7.53	7.43	9.09	5.80	e_2
666	9.69	10.70	9.22	8.39	9.21	e_2
710	3.52	12.68	9.24	2.21	6.20	e_2
740	10.26	11.43	11.27	7.96	9.25	e_1
841	10.52	14.30	8.25	7.60	11.19	e_1
851	10.37	11.73	9.90	9.06	13.78	e_1
922	11.87	12.54	11.38	8.69	13.98	e_1

Table 2: Estimated damping B for each participants, according to the experimental conditions

**M** (kg)						
	Experiment P			Experiment H		
User ref	φ_1	φ_2	φ_3	h_1^* (φ_1)	h_3^* (φ_1)	Performance group
000	0.349	0.374	0.413	0.364	0.332	e_3
008	0.397	0.337	0.331	0.487	0.442	e_2
029	0.581	0.455	0.585	0.523	0.460	e_1
041	0.603	0.375	0.472	0.648	0.599	e_3
050	0.583	0.446	0.522	0.489	0.395	e_1
113	0.410	0.417	0.469	0.424	0.391	e_2
160	0.548	0.507	0.680	0.431	0.542	e_1
194	0.505	0.328	0.370	0.472	0.521	e_2
208	0.328	0.306	0.321	0.330	0.350	e_1
211	0.545	0.324	0.352	0.414	0.406	e_2
278	0.508	0.433	0.478	0.544	0.515	e_1
311	0.299	0.302	0.345	0.369	0.331	e_2
353	0.364	0.376	0.534	0.541	0.432	e_1
364	0.422	0.450	0.531	0.479	0.506	e_2
378	0.483	0.511	0.508	0.452	0.401	e_3
388	0.259	0.252	0.335	0.285	0.272	e_2
418	0.363	0.272	0.412	0.440	0.519	e_2
456	0.326	0.229	0.293	0.300	0.332	e_1
495	0.263	0.227	0.270	0.300	0.292	e_2
546	0.413	0.313	0.365	0.436	0.481	e_1
548	0.472	0.469	0.537	0.382	0.406	e_3
555	0.592	0.516	0.539	0.501	0.491	e_2
573	0.572	0.404	0.482	0.665	0.549	e_3
640	0.302	0.272	0.412	0.438	0.371	e_1
661	0.326	0.295	0.356	0.297	0.306	e_2
666	0.524	0.427	0.535	0.498	0.449	e_2
710	0.815	0.698	0.688	0.893	0.680	e_2
740	0.294	0.258	0.381	0.369	0.361	e_1
841	0.571	0.423	0.532	0.409	0.392	e_1
851	0.635	0.533	0.567	0.456	0.486	e_1
922	0.417	0.316	0.416	0.599	0.458	e_1

Table 3: Estimated mass M for each participants, according to the experimental conditions

R^2						
	Experiment P			Experiment H		
User ref	φ_1	φ_2	φ_3	$h_1^* (\varphi_1)$	$h_3^* (\varphi_1)$	Performance group
000	0.980	0.974	0.971	0.981	0.979	e_3
008	0.965	0.969	0.973	0.943	0.913	e_2
029	0.898	0.962	0.942	0.958	0.953	e_1
041	0.939	0.968	0.968	0.886	0.938	e_3
050	0.922	0.963	0.960	0.917	0.945	e_1
113	0.930	0.976	0.933	0.922	0.953	e_2
160	0.907	0.963	0.930	0.955	0.895	e_1
194	0.925	0.954	0.986	0.916	0.917	e_2
208	0.973	0.974	0.966	0.949	0.963	e_1
211	0.890	0.967	0.963	0.957	0.922	e_2
278	0.944	0.976	0.968	0.958	0.949	e_1
311	0.951	0.983	0.975	0.943	0.921	e_2
353	0.928	0.980	0.963	0.919	0.830	e_1
364	0.920	0.959	0.975	0.949	0.948	e_2
378	0.934	0.982	0.967	0.952	0.949	e_3
388	0.969	0.968	0.975	0.982	0.951	e_2
418	0.934	0.976	0.969	0.953	0.896	e_2
456	0.963	0.970	0.977	0.957	0.963	e_1
495	0.958	0.947	0.961	0.926	0.966	e_2
546	0.912	0.979	0.945	0.905	0.947	e_1
548	0.934	0.953	0.953	0.944	0.895	e_3
555	0.943	0.979	0.956	0.949	0.899	e_2
573	0.908	0.977	0.974	0.934	0.928	e_3
640	0.959	0.976	0.947	0.960	0.957	e_1
661	0.961	0.964	0.970	0.962	0.953	e_2
666	0.893	0.940	0.928	0.885	0.906	e_2
710	0.889	0.933	0.920	0.918	0.902	e_2
740	0.980	0.976	0.982	0.945	0.900	e_1
841	0.894	0.969	0.965	0.947	0.893	e_1
851	0.942	0.963	0.958	0.913	0.944	e_1
922	0.939	0.981	0.977	0.826	0.935	e_1

Table 4: Average restitution coefficient of the reconstructed position trajectories with the estimated parameters

<εr> (cm)				
User ref	Exp. P (h_2^*)	Exp. H (h_1^*)	Exp. H (h_3^*)	Performance group
000	1.69	4.44	2.70	e_3
008	2.78	8.05	2.48	e_2
029	10.57	3.34	6.79	e_1
041	7.85	4.28	4.39	e_3
050	13.77	12.28	11.15	e_1
113	0.32	3.24	-0.21	e_2
160	3.03	8.37	-3.03	e_1
194	8.49	17.17	2.30	e_2
208	-4.89	-2.75	-5.97	e_1
211	6.04	6.69	6.77	e_2
278	14.86	15.08	7.91	e_1
311	6.77	13.12	10.80	e_2
353	4.10	6.93	0.24	e_1
364	-2.12	3.70	-4.50	e_2
378	6.93	7.37	8.34	e_3
388	7.68	9.00	-0.13	e_2
418	-0.12	-2.87	-5.24	e_2
456	17.26	24.05	14.85	e_1
495	6.68	2.91	1.70	e_2
546	7.45	11.94	4.00	e_1
548	5.69	6.75	0.67	e_3
555	2.57	-1.18	-6.51	e_2
573	1.69	-1.84	-1.14	e_3
640	3.94	14.71	6.27	e_1
661	7.12	12.18	7.84	e_2
666	2.77	1.17	-4.45	e_2
710	-5.11	-3.64	-10.10	e_2
740	7.69	7.85	5.96	e_1
841	5.15	6.11	25.33	e_1
851	8.77	4.73	6.78	e_1
922	8.58	8.22	4.70	e_1

Table 5: Precision, estimated with the average bouncing error, for each participants, according to the experimental conditions

$\sigma(\varepsilon_r)$ (cm)				
User ref	Exp. P (h_2^*)	Exp. H (h_1^*)	Exp. H (h_3^*)	Performance group
000	17.07	14.24	15.10	e_3
008	21.36	21.28	23.82	e_2
029	28.16	19.49	27.72	e_1
041	21.68	15.66	18.57	e_3
050	26.26	20.64	29.06	e_1
113	20.25	22.03	20.70	e_2
160	26.22	24.12	26.29	e_1
194	23.20	19.76	19.59	e_2
208	26.34	21.71	26.46	e_1
211	26.05	16.57	31.21	e_2
278	28.68	29.15	36.81	e_1
311	18.74	20.61	24.35	e_2
353	26.65	23.45	31.31	e_1
364	23.03	20.85	25.58	e_2
378	18.12	17.18	25.96	e_3
388	21.73	22.26	21.86	e_2
418	21.62	24.73	30.73	e_2
456	33.58	33.14	43.57	e_1
495	24.51	21.29	25.75	e_2
546	29.59	25.13	34.53	e_1
548	16.89	16.68	21.37	e_3
555	21.59	18.52	26.01	e_2
573	17.37	15.88	21.87	e_3
640	21.68	28.60	33.95	e_1
661	25.72	18.86	27.12	e_2
666	24.74	24.97	23.20	e_2
710	22.72	21.42	26.31	e_2
740	23.56	23.40	35.34	e_1
841	34.85	23.32	36.42	e_1
851	28.00	25.87	26.85	e_1
922	28.48	22.32	25.75	e_1

Table 6: Repeatability estimated using the standard deviation of the bouncing error, for each participants, according to the experimental conditions
