# Supplementary material for: Human arm endpoint-impedance in rhythmic human-robot interaction exhibits cyclic variations
Source: PLoS One. 2023 Dec 14;18(12):e0295640. doi: 10.1371/journal.pone.0295640 (PMC10721195; doi:10.1371/journal.pone.0295640)
Supplement: Attachment — Submitted filename: Response to Reviewers.pdf [file pone.0295640.s002.pdf]

Response to Reviewers

Dear Editor-in-Chief,

We are grateful for the reviews provided by the editors and each of the reviewers. Please find below our detailed response to the reviewers comments. Line numbers refer to the manuscript with tracked changes (the line numbering is different from the document without tracked changes), some of these changes were included in blue in this document. We made few changes to the introduction and added one short paragraph to the discussion accordingly. The manuscript English was revised by a professional.

Reviewer #1:

R1.1. Reviewer comment: *The first part of the Introduction section describes the idea, which I would briefly summarize as "the models should be as simple as they can be but not simpler". This idea is, however, presented in a convoluted manner. After introducing the utility of "simple mechanical models", the description delves into specifics and describes "detailed models". Then, it returns to the "simple models", narrating specific locomotive CPG-based models afterward. This type of flow is often undesirable. I would suggest following a generally recommended format (often called "the inverted pyramid") of the Introduction section: (1) introduce essential information, (2) provide important details or supporting information, and (3) list even more specific details.*

Authors' response: The introduction has been reworked, with a structure close to the inverted pyramid.

R1.2. Reviewer comment: *The Introduction section also needs to include the essential details readers want to know without going through references. For example, line 35 refers to "dynamic limbs' properties" without stating them explicitly; line 37 states "13 parameters" are necessary to account for viscoelastic properties of the muscles but does not hint what the nature of those parameters is.*

Authors' response: More details have been provided for the model with 13 parameters from line 40 to 44 in the revised manuscript. "To account for passive muscle viscoelastic properties and active behaviors induced by reflexes and motion, Latash and Zatsiorsky [14] explained that a single joint would at least need 13 parameters. They considered two delays for the viscoelastic contribution of both the short-latency monosynaptic reaction and the tonic stretch reflex, with their respective stiffness and damping."

R1.3. Reviewer comment: *Figures require a more detailed description. For example, although the Figure 1 is self-explanatory, it may be not easy to understand to a first-time reader. I would suggest expanding basic figure descriptions in lines 105, 121, 135, 157, and 223.*

Authors' response: The figure subtitles have been detailed more thoroughly.

R1.4. Reviewer comment: *It is imperative to define abbreviations. In line 32, the abbreviations "KBM" and "KBI" are not previously described. Throughout the text, it becomes clear that K denotes stiffness, B stands for damping, and M is used for mass. However, it is essential that the reader is not forced into guessing and focuses on science rather than deciphering "KBM", "KBI", and, perhaps, "ARX" in line 159.*

Authors' response: The abbreviations KBM (lines 46-47) and ARX "(Auto Regressive model with eXternal inputs)" (line 238-239) have been explicated.

R1.5. Reviewer comment: *The terms of the equation (lines 164-165) are not explained. The description in lines 160-164 could be expanded to clarify how the equation was obtained. The term ∂f (delta f) is ambiguously defined in line 140. Perhaps, the wrong subscript (or the absence of an appropriate superscript) was used to describe the perturbation force.*

Authors' response: The terms of equation (2) and (3) have been detailed. ∂f is the differential force that is generated by the perturbation. The subscript typos have been corrected (lines 217-218). "This model relates a differential force δf to a differential position δx when a perturbation force (equal to δf) is introduced along the virtual trajectories f_0 and x_0." And the undefined terms of equation (3) have been added (lines 239-243). "The estimation on a discretized impedance model, with q^{-1} the delay operator and e white noise [...] The relationships between the discrete-time coefficients (a_i and b_i) and the continuous-time impedance parameters K, B and M are given in (4). They can be obtained with a zero order hold discretization with a time step of Δt equal to the sampling time of 1 ms."

R1.6. Reviewer comment: *The "Data Analysis" subsection (line 181) describes the use of a k-means algorithm to cluster 31 participants into three performance groups: (1) novice, (2) intermediate, and (3) advanced. It is unclear if the number of groups (clusters) was determined automatically. Could you please explain how you selected the number of groups? In other words, why was the number of groups (centroids in k-means) equal to three and not two? Unless additional custom computations are performed on top, the k-means algorithm runs optimization to distinguish between a predefined number of groups regardless of the dataset you pass. The k-means can also overfit the data. It is therefore necessary to explain the choice of the number of groups.*

Authors' response: The number of groups of the k-means algorithm has been determined by the authors themselves, since it was not obvious in the paper, it has been clarified. We tested the clustering on 2, 3 and 4 groups.

Number of clusters	2	3	4
Sum of quadratic distance to centroid	0.0248	0.0147	0.0070
Group repartition (from experts to beginners)	15 / 16	5 / 13 / 13	5 / 11 / 10 / 5

Interestingly the expertise group is the same for 3 and 4 clusters, and the intermediate groups are almost the same (only 2 users are taken out). Therefore, the main difference of the k-means algorithm for 3 and 4 clusters is the splitting of the beginner group into 2 different groups. This is why the authors have chosen to use 3 groups.

R1.7. Reviewer comment: *The statement about the significant/insignificant results obtained on the same data with Holm/Bonferroni corrections need to be either clarified or removed.*

Authors' response: The fact that the Holm correction reveals significant variations while not the Bonferroni correction might indicate a False negative from the Bonferroni correction. This has been added line 439. "The couple (φ_1, φ_2), indicated a significant variation with Holm correction but not with Bonferroni correction, which might reveal a false negative of the Bonferroni correction."

R1.8. Reviewer comment: *Throughout the paper, SD used to abbreviate standard deviation. However, the standard deviations are used inconsistently. For example, the line 257 uses "(6.3 SD)" notation, while the line 258 uses "(SD 4.2)". Other examples exist throughout the paper.*

Authors' response: The notation SD has been corrected (line 387).

R1.9. Reviewer comment: *The statements in lines 250-253 may not be clear. The paragraph states that the participants hit the ball at similar vertical positions, listing the ball-bouncing magnitudes that are ~40% different from each other. I might have misunderstood the authors. Clarifications are needed.*

Authors' response: The impact position is equal to the target heights minus the ball bouncing magnitude. The impact height has been added for a more fluid comprehension lines 382-383. "(0.324 m for h1* and 0.330 m for h3*)".

R1.10. Reviewer comment: *The table data are accessible, however the raw data and the computer code for data processing and analysis were not attached.*

Authors' response: The raw data is quite heavy (>90 Go). We can provide a snippet for the data processing, with a sample of raw data if required.

R1.11. Reviewer comment: *The manuscript requires heavy editing. It has multiple grammar, stylistic, and punctuation errors. For the next submission, I suggest using error correcting software (e.g., Grammarly).*

Authors' response: The paper has been proofread by a professional.

Reviewer #2:

R2.1. Reviewer comment: *The paper's aim is presented shortly in the introduction (last paragraph, lines 75-80). It should be elaborated, allowing readers to understand this study's*

purpose. It is somehow highlighted in the discussion part, where authors compare their results to ones given in the literature.

Authors' response: The main purposes and contributions of the study have been added to the introduction (lines 117-120). "The results presented in this paper contribute mainly to three questions: 1) "Are there endpoint-impedance variations during a cyclic task, if yes, what is the nature of these variations?", 2) "Is there a relation between the endpoint-impedance behavior and the constraints of the task?", and finally 3) "Is there a link between the endpoint-impedance parameters and the performances reached?"."

R2.2. Reviewer comment: *The experimental stand is described briefly in the "Apparatus" subsection. It is accompanied by a simple drawing (Fig. 1). Please, provide a detailed photo of the experimental setup indicating the hardware used.*

Authors' response: The drawing describing the experimental setup has been elaborated with a simulated environment, however, no decent photograph can be properly used for the setup description.

R2.3. Reviewer comment: *Authors briefly describe the ball-bouncing task. However, the simulation environment needs to be described. Understanding the interaction between the participant, robot, and simulated environment would be crucial. Moreover, it would give better insights into data collection for further analysis.*

Authors' response: The simulation was achieved using ROS and RViz. As added lines 307-308, the data from the simulated environment was not used for the impedance estimation. And more details about the simulation are provided lines 163-164. "The uni-dimensional task was displayed on a screen, with the ball represented by a sphere (mass: 0.058 kg, radius: 0.0325 m) and the paddle by a bar (mass: 1 kg)."

R2.4. Reviewer comment: *The parameters of equation (1) should be properly described, e.g. is 'k' is a moment of the impact or discrete time? What is the parameter 'alpha'?*

Authors' response: Equation (1) notation has been modified for readability, and alpha is a restation coefficient as added line 188-189. "[…] α the same restitution coefficient used for the ballistic kinematics".

R2.5. Reviewer comment: *In Fig. 2, the 'arm position' is given in [cm] and 'ball position' in [m]. It raises the question if the estimated parameters of the participant movement model are correct. Explain if there exists/or not exists the possible scaling effect, as the scale of the perception of the task performed (ball on the screen) and the real task performance (movement of a robot arm) can play a role in the estimation of the parameters. In such a case, the participant's height or body posture can be a significant factor.*

Authors' response: The Fig. 2 mixes both units just to avoid having floating numbers that are more difficult to read. There is a scaling effect between the real movement operated by the robot, and the movement in the simulation. The proportional scale is the same for every

participant and was chosen to keep the simulation doable, as added in the ball-bouncing task section (lines 161-163). “The simulation was calibrated so that the participants were able to approach performances close to the ones described in [27,28] to guarantee rhythmic movements.” The task achieved by the participant is indeed not a real bouncing task, it is rather a video game with an haptic interface.

R2.6. Reviewer comment: *The ARX identification method should be described in detail.*

Authors’ response: Much more details have been provided for the ARX identification and the estimation of the continuous impedance parameters lines 239 to 243, and equation (4) provides the relationship between the continuous and discrete parameters. “The relationships between the discrete-time coefficients (a_i and b_i) and the continuous-time impedance parameters K, B and M are given in (4). They can be obtained with a zero order hold discretization with a time step of Δt equal to the sampling time of 1 ms.”

R2.7. Reviewer comment: *The parameters and the variables in equation (2) are not described.*

Authors’ response: May be the reviewer was referring to equation (3) which has been detailed, since it was already pointed out by the other reviewer (**R1.5**).

R2.8. Reviewer comment: *The experiments are indicated with the letters "P" and "H". Please, describe the meaning of these letters.*

Authors’ response: The experiments are indicated with the letters "P" for phase and "H" for height, as added line 288.

R2.9. Reviewer comment: *In the Discussion part, the authors write about the results obtained for estimating the KBM model parameters (stiffness, damping, mass). The estimation method must be described properly in the final version of the manuscript.*

Authors’ response: The estimation method has been further detailed in the ARX section lines 238 to 243, with equation (4) line 284. (see **R2.6**)
