# Supplementary material for: Human arm endpoint-impedance in rhythmic human-robot interaction exhibits cyclic variations
Source: PLoS One. 2023 Dec 14;18(12):e0295640. doi: 10.1371/journal.pone.0295640 (PMC10721195; doi:10.1371/journal.pone.0295640)
Supplement: Attachment — Submitted filename: Response to Reviewers.pdf [file pone.0295640.s003.pdf]

Response to Reviewers

Dear Editor-in-Chief,

We are grateful for the reviews provided by the editors and each of the reviewers. Please find below our detailed response to the reviewers' comments. Line numbers refer to the manuscript with tracked changes (the line numbering is different from the document without tracked changes), some of these changes were included in blue in this document. We made few changes to the introduction to try to improve its readability and made another pass of proofreading using the recommended tools.

Reviewers' comments:

The main concerns were about 1) the statistical analysis, and 2) grammatical errors.

In response to the reviewers' comments, we have carefully revised the manuscript. Below, we provide a point-by-point response to each of the reviewers' comments:

Reviewer #1:

R1.1. comment: The reworked statement about the significant/insignificant results reveals that the authors need to review their statistics again. The description for the use of Holm or Bonferroni corrections does not explain 'why' both were used. If the authors want to include the results of both methods in the paper, the rationale for using them should be described. For example, if it is critical to avoid Type I errors (false positives) at all costs, even if it means potentially missing some true effects, then the Bonferroni correction would be appropriate. If you want to balance the trade-off between avoiding false positives and not missing true effects, then Holm's method would be a better choice, as it offers more power while still controlling the family-wise error rate.

Authors' response: Since we want our results to be reliable, we rather keep the Bonferroni correction. Therefore, the statement about a possible missing true effect was deleted.

R1.2. comment: The data must be accessible for reviewers to validate the truthfulness of the figures.

Authors' response: We made accessible the JASPs files used for the statistical analysis and the post-processed cyclic data, the pure raw data is too heavy and complex to exploit.

R1.3. comment: The authors stated a professional reviewed the text. This effort was insufficient. If grammatical errors are difficult to spot, please use free grammar correction software (e.g., Grammarly or others). PLOS ONE guidelines disallow me to pass the manuscript unless it is error-free.

Authors' response: Another pass was done using AI tools. We must admit that the quality of the previous proofreading was indeed questionable, we are sorry for the inconvenience.

R1.4. comment: There was an improvement in the Introduction, thank you. Still, it remains unclear. The sentences are disconnected and convoluted (the reader cannot follow your argument). The first paragraph of the section, for example, is unreadable. Wordy sentences disservice your paper and should be avoided in favor of clear explanations.

Authors' response: We tried to improve the readability of the introduction.
